# Expanding the phenotypic spectrum associated with *CFAP43* mutations: a case report of familial male infertility with respiratory manifestations

**DOI:** 10.3389/frph.2025.1609938

**Published:** 2025-11-18

**Authors:** Fawaz Awad, Razan Abukhaizaran, Shahira Al Jabi, Mustafa Nabilsi, Laith A. Ayasa, Majd A. AbuAlrob, Haneen Owienah, Hanin Kassem, Moien Kanaan

**Affiliations:** 1 Department of Molecular Genetics Laboratory, Istishari Arab Hospital, Ramallah, Palestine; 2 Molecular Genetics and Microbiology Research Laboratory, Faculty of Medicine, Al-Quds University, Jerusalem, Palestine; 3 Razan Centre for Infertility, Ramallah, Palestine; 4 Department of Radiology, Istishari Arab Hospital, Ramallah, Palestine; 5 Molecular Genetic Lab, Bethlehem University, Bethlehem, Palestine

**Keywords:** *CFAP43*, male infertility, asthenoazoospermia, multiple morphological abnormalities of the flagella, primary ciliary diskynesia

## Abstract

**Aims:**

Multiple morphological abnormalities of the sperm flagella (MMAF) represents a rare and severe form of male infertility, characterized by defects in sperm flagella. Mutations in genes essential for flagellar function, such as *CFAP43*, have been implicated in MMAF. Flagella and motile cilia share a conserved axonemal structure essential for their motile function and the asthenospermia-related infertility of MMAF overlaps with primary ciliary dyskinesia (PCD) symptoms, characterized by chronic airway disease and infertility due to ciliary and flagellar dysfunction. This study investigates the genetic basis of MMAF in two siblings, who also exhibited respiratory symptoms.

**Methods:**

Clinical assessment and semen analysis were conducted for two brothers presenting with infertility and chronic respiratory symptoms. Whole-exome sequencing (WES) was performed to identify potential genetic defects.

**Results:**

Both siblings exhibited classic MMAF features, including asthenospermia with various flagellar abnormalities, in addition to chronic respiratory symptoms including sinusitis and wet cough. WES identified a novel homozygous missense genetic variation in *CFAP43* [c.421T>A p.(Trp141Arg)].

**Conclusion:**

Our findings provide additional evidence of the genetic contribution of *CFAP43* in MMAF and suggest an expanded phenotypic spectrum of *CFAP43*-associated conditions to encompass chronic respiratory symptoms attributed to airway ciliary dysfunction. Further research is needed to uncover the underlying mechanisms linking *CFAP43* mutations to these phenotypes.

## Introduction

Male infertility is a rapidly growing concern, contributing to around 50% of infertility cases. While the revolutionary technique of intracytoplasmic sperm injection (ICSI) has been a treatment option for many cases of male infertility, the aetiology of male infertility remains elusive in nearly 50% of cases (1). A shift towards identifying the underlying causes of infertility and exploring advanced personalized management have gained momentum. This endeavour has been aided by advanced genetic testing, which has uncovered several previously unknown genetic conditions contributing to infertility.

Multiple morphological abnormalities of the sperm flagella (MMAF) is a rare yet severe cause of asthenospermia-related infertility, where sperm flagella show a wide range of malformations, including short, coiled, absent, and irregular flagella forms leading to impairment in sperm motility and morphology, two critical factors in successful fertilization and normal reproductive function. These abnormalities are primarily a consequence of alterations in the ultrastructure of the flagellar axoneme, which can include defects in the highly organized microtubules or proteins involved in their assembly, as well as the surrounding dense fibers (2).

The axoneme, typically organized in a ″9 + 2″ microtubule arrangement, consists of nine microtubule doublets surrounding a central pair of microtubules. This structure is crucial for the motility of both sperm flagella and motile cilia. The microtubule doublets serve as a structural scaffold, supporting the movement, while the central pair complex stabilizes and coordinates movement through radial spokes. These spokes help transmit signals for synchronized motion. The dynein arms, motor proteins on the outer doublets, generate the force needed for axonemal bending by moving along adjacent microtubules (2).

The use of high-throughput sequencing techniques such as whole exome sequencing (WES) has significantly advanced the genetic diagnosis of MMAF. About 43 MMAF-associated genes have so far been identified, with mutations in the *DNAH1*, *CFAP43*, and *CFAP44* genes responsible for approximately one-third of MMAF cases (3). However, the molecular mechanisms linking these defects to the MMAF phenotype are still largely poorly understood. Additionally, the exact relationship of these defects with patients' phenotypes is not well established.

Cilia and flagella have similar axonemal structure sharing many essential constitutional proteins. This can be clinically manifested in PCD, in which patients have manifestations of ciliary dysfunction, including chronic airway disease and, in most cases, infertility due to flagellar dysfunction. Nevertheless, it is noteworthy that cilia and flagella may have differences in the molecular associated function indicated by the fact that not all PCD patients experience infertility, and that most individuals with MMAF do not present respiratory symptoms (4).

Recently, biallelic loss-of-function mutations in the *CFAP43* gene (cilia- and flagella-associated protein 43, MIM#: 617592), which encodes a protein necessary for normal sperm flagella axonemal organization, were reported as a causative factor of MMAF in patients with infertility (5, 6). Since then, about 29 *CFAP43* MMAF-causing genetic variations (pathogenic or likely pathogenic) have been described in the literature (7). However, none of the described patients showed symptoms related to defects with airway cilia function. It is worth noting that a nonsense variation in the *CFAP43* gene has been linked to adult-onset normal-pressure hydrocephalus, suggesting that this gene may also be involved in the functioning of cilia in the ventricles of the brain (8).

In this study, we aimed to elucidate the molecular basis of MMAF-induced male infertility in a Palestinian family with two affected siblings who exhibited chronic respiratory symptoms indicative of ciliary dysfunction of the airways. We conducted WES in the two siblings and identified a novel missense variation in the *CFAP43* gene.

## Method

### Patients

Two male brothers diagnosed with MMAF referred from Razan Centre for Infertility (Palestine) were enrolled in this study. Both patients sought infertility treatment from the years 2005 to 2007. They were enrolled in this study for genetic diagnosis and evaluation of respiratory symptoms. A standardized clinical information form was filled out for each patient.

### Semen analysis

Semen samples were collected via masturbation following 2–7 days of sexual abstinence. The samples were examined after liquefaction for 30 min at 37°C. Semen analyses were repeated twice for each individual six weeks apart. Semen analysis, including morphological assessment of sperm, was performed per the 4th edition of the WHO manual.

### Genetic investigations

Molecular diagnosis was performed at the molecular genetic laboratory of the Istishari Arab Hospital (Ramallah, Palestine). Genomic DNA was extracted from 5 ml peripheral blood samples collected from both patients and an apparently healthy unrelated control. WES was performed using the TruSeq Capture Exome Kit (Illumina), targeting 45 Mb of exonic content to obtain at least 20× coverage depth for >98% of the targeted bases. Sequencing was conducted on the NextSeq500 platform, and data were aligned to the human genome reference (hg19) using the BWA aligner and ANNOVAR was used for variant annotation. Copy-number variants (CNVs) were called using an in-house bioinformatics CNV detection pipeline.

A stepwise filtering approach was applied to prioritize variants relevant to the phenotype (Supplementary Figure 1). Variants with a read depth <5 were excluded, and common variants were filtered out based on a minor allele frequency (MAF) threshold of >1% in gnomAD (v2.1.1) and >5% in our in-house exome/genome database. The analysis focused on coding exons and flanking +/−10 intronic nucleotides. Variants predicted to have a high or moderate impact on protein function including missense, nonsense, frameshift, and splice site variations were included. Synonymous and intronic variants beyond 10 nucleotides from exon-intron boundaries were excluded unless they were known to affect splicing or previously linked to disease. Remaining variants were evaluated for pathogenicity using prediction tools such as PolyPhen-2, SIFT, AlphaMissense, and CADD. Following this filtration process, a final list of candidate variants was generated (Supplementary Table 1). The *CFAP43* variant identified through WES was subsequently confirmed by Sanger sequencing using the following primers for PCR amplification and sequencing: F: 5′-ACTTGGACTTGATAAGCACAGC-3′; R: 5′-GCCTTGGTATAGCATATGACAGT-3′.

## Results

### Clinical presentation of MMAF patients

The two patients (Patient 1 and Patient 2) are siblings born to a consanguineous union (first-degree cousins) from a village in the West Bank (Palestine) who both consulted for primary male infertility. Semen analysis of both patients revealed normal sperm count. In contrast, an almost complete asthenospermia with the absence of progressive sperm motility was observed in both patients. In addition, very few spermatozoa with normal morphology were present (1% and 2% respectively) (Table 1). An initial diagnosis of MMAF was established based on the following criteria: Infertility in addition to the presence of spermatozoa with 5 morphological abnormalities: short, absent, coiled, bent, and irregular flagella. None of the siblings reported exposure to gonadotoxic factors.

**Table 1:** Clinical and semen characteristics of the individuals with the identified CFAP43 genetic variation.

Individual	Patient 1	Patient 2	
Age	54	51	
Neonatal distress	N	N	
Chronic sinusitis	Y	Y	
Chronic wet cough	Y	Y	
Otitis media	N	N	
Bronchiectasis	N	Focal	
Situs Inversus	N	N	
Infertility	Y	Y	
Semen characteristics			Reference Limits
Sperm Volume (ml)	4.2	5	>1.4
Total Sperm Count (10^6^/ejaculate)	52.5	100	≥39
Progressive Motility (%)	1	0	≥30
Morphology (% typical forms)	1	2	≥4

N, No; Y, Yes.

**Figure 1:**
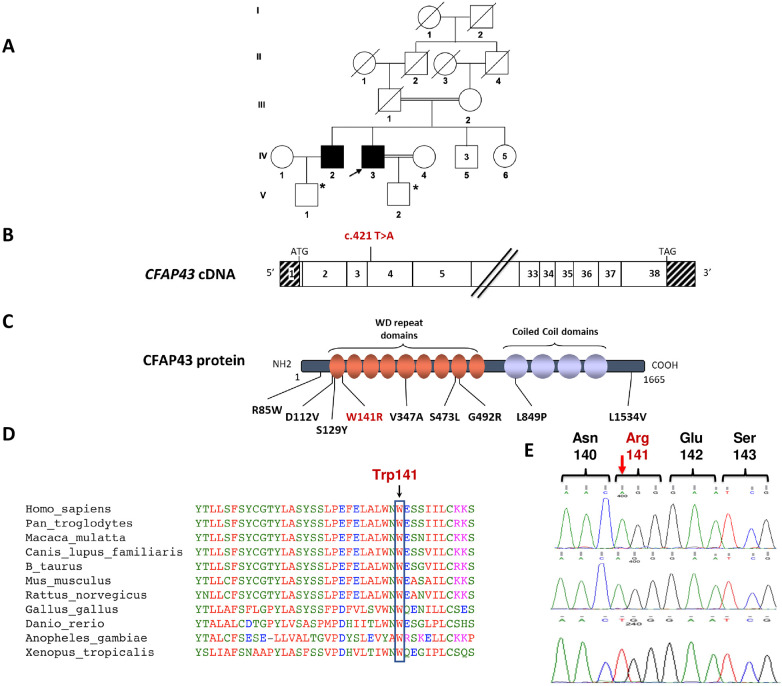
Identification of the homozygous c.421T>A; p.(Trp141Arg) missense variation in the *CFAP43* gene. **(A)** Genealogical tree of the studied family. The black arrow shows the Proband. *Products of assisted reproductive technology through ICSI. **(B)** Exonic organization of the human *CFAP43* cDNA in which the genetic variation described in this study is shown. The exons of *CFAP43* are indicated by empty or hashed boxes depicting translated or untranslated sequences, respectively. **(C)** Domain-organization model of the CFAP43 protein as described by Uniprot server with a summary of previously reported MMAF-associated missense variations (in black font) and the novel variation identified in this study (in red font). **(D)** A partial protein sequence alignment of *CFAP43* among various species shows the evolutionary conservation of the amino acid substitution identified in this study. Sequence alignment shows that Trp141 is a highly conserved residue. **(E)** Sanger sequencing confirmed the presence of the genetic variation (c.421T>A) in *CFAP43*, which was identified by WES in the two brother patients (upper and middle electropherograms). The sequence of the identified *CFAP43* variation locus is also shown in a healthy control, showing the wild-type (WT) genotype (lower electropherogram).

Both patients are married and have a son each via assisted reproductive technology. Patient 1 (Figure 1A, IV2), who is now 54 years old, has a history of 8 ICSI trials, and one of them was successful. Patient 2 (Figure 1A, IV3), who is now 51 years old, also has a history of 4 ICSI trials, and one of them was successful. As per the genealogical tree shown in Figure 1A, they have 3 brothers and 5 sisters, all of them had naturally conceived infants.

**Figure 2:**
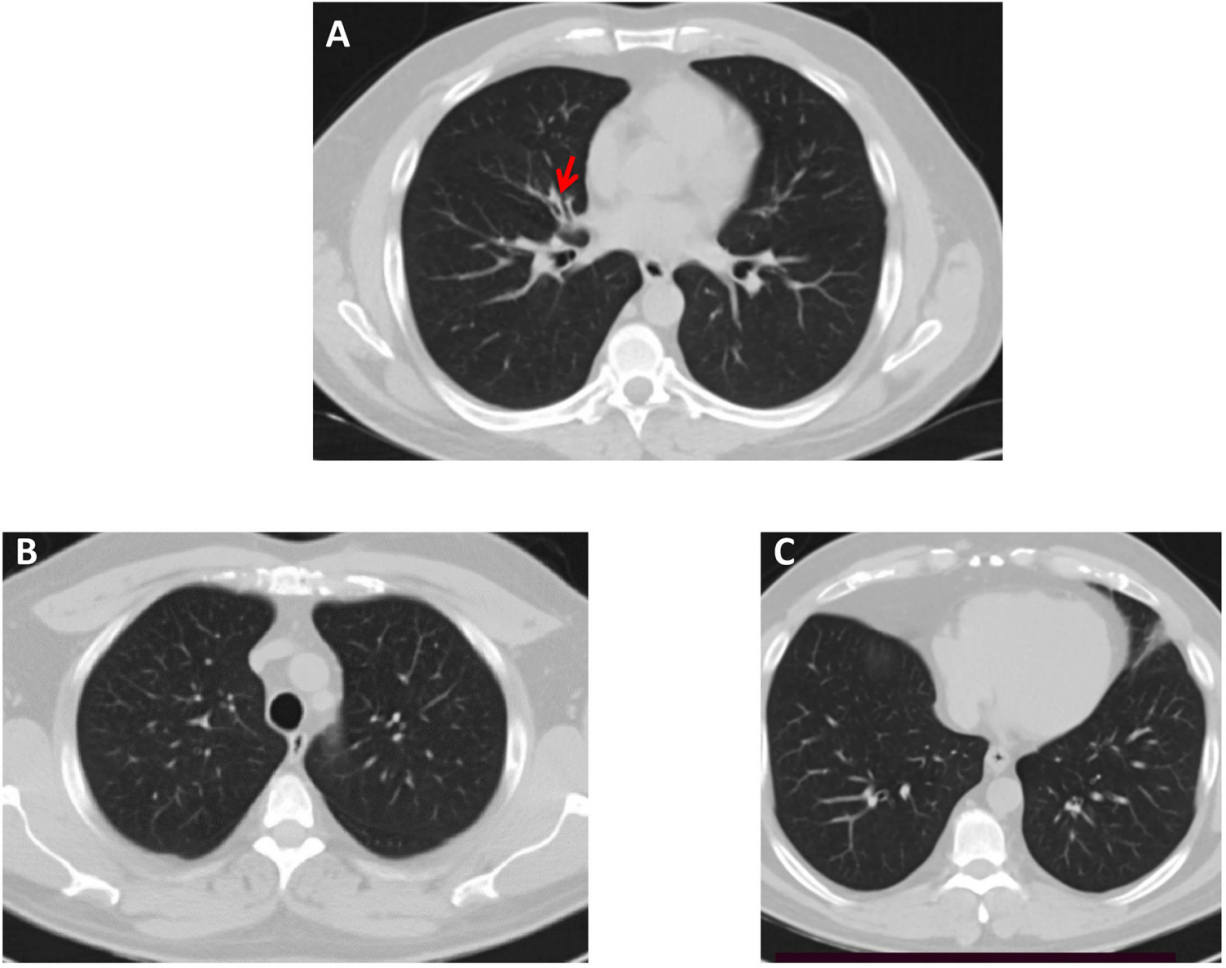
High-resolution chest CT scan—lung window, of Patient 2. **(A)** A small area of bronchiectasis in the medial segment of the right middle lobe, indicated by a red arrow. **(B,C)** Both lung fields in the upper and lower lobes appear normal.

Importantly, both patients reported that they also suffer from respiratory symptoms characterized by chronic sinusitis and chronic wet cough since infancy (Table 1) and therefore, they were referred to a pulmonology clinic for further evaluation and follow-up. As shown in Figure 2, a chest computerised tomography (CT) scan of patient 2 revealed a localized area of bronchiectasis in the medial segment of the right middle lobe of the lung. Moreover, cystic bronchiectasis accompanied by mucus plugging was also observed, particularly in the lower lobes. There were no signs of laterality defects in the CT scan, which was expected based on the patient's medical history. Patient 1 refused to be evaluated for the respiratory symptoms.

### Identification of a missense variation in the *CFAP43* gene

To identify the underlying genetic cause of this condition, we performed WES for both affected siblings. WES analysis led to the identification of a homozygous c.421T>A p.(Trp141Arg) variant in exon 4 of the *CFAP43* gene in both patients (Figure 1B). The allele frequency of this variant was extremely low in gnomAD and was absent from 1,000 healthy ancestry-matched control samples. Additionally, the identified sequence variation is predicted to be deleterious according to SIFT, PolyPhen-2, AlphaMissense (score = 0.987) and CADD (Phred score = 27.4) (Supplementary Table 1). This variant leads to amino acid substitution at codon 141 involving the amino acid tryptophan that is conserved throughout evolution and located within the WD repeat domains of the CFAP43 protein (Figures 1C,D). No variants have been previously described as disease-causing in the literature or reported in clinical variant databases at the same nucleotide (c.421T) or at adjacent nucleotides affecting the same codon that would result in an identical or different amino acid substitution. The identified *CFAP43* variation was further verified by Sanger sequencing in both patients (Figure 1E). Unfortunately, family-based segregation analysis could not be performed due to the unavailability of DNA samples from the parents and healthy siblings of the patients.

To rule out other potential genetic contributors to the phenotype, we reviewed all variants identified in known MMAF and PCD genes, irrespective of their predicted pathogenicity (Supplementary Table 2). Each variant underwent assessment and was classified as benign or likely benign, or found in a monoallelic state (one heterozygous variant) in genes with a recessive mode of inheritance, thus ruling out their involvement in the patients' phenotype. These findings collectively suggest that the c.421T>A p.Trp141Arg missense variation in *CFAP43* is causative for the MMAF phenotype and associated PCD-like symptoms in this family.

## Discussion

In this study, we investigated two brothers diagnosed with MMAF associated with chronic respiratory manifestations, both carrying a homozygous variation [c.421T>A p.(Trp141Arg)] in the *CFAP43* gene. Our findings provide the first evidence of a *CFAP43* genetic variation causing asthenospermia-related infertility in Palestine. Additionally, they highlight the potential expansion of the *CFAP43* gene's phenotypic spectrum to include respiratory manifestations.

Although direct evaluation of the functional and structural impact of the p.(Trp141Arg) missense variation on patients' spermatozoa was not feasible due to the lack of fresh biological samples, we postulate its causality based on similarities observed in the semen analysis of our patients compared to previously reported patients with *CFAP43* mutations (6). Furthermore, several lines of molecular evidence support the potential pathogenicity of the identified *CFAP43* variation. Firstly, it resides in a critical hotspot within the WD repeat domains, where most reported pathogenic missense *CFAP43* variations are found (Figure 1C) (7). Secondly, the evolutionary conservation of the Trp141 residue underscores its significance in the biological role of the CFAP43 protein. Additionally, the identified p.(Trp141Arg) variation in CFAP43 protein is predicted to be deleterious using mutation-prediction bioinformatic tools such as SIFT, Polyphen-2 and CADD.

While most previously identified *CFAP43* variations are loss-of-function truncating mutations (frameshift, splicing, or stop mutations), our findings reveal a novel missense variation p.(Trp141Arg), that needs to be assessed by functional studies. Interestingly, previous reports have indicated that missense variants in *CFAP43* are associated with milder symptoms manifested by mild asthenospermia (5). However, this was not observed in the studied family, as both siblings suffered from almost complete asthenospermia in addition to the chronic respiratory symptoms.

Several studies have reported genetic variations in *CFAP43* as a causative factor for MMAF in infertility patients, and this was further confirmed by functional studies and various knockout mouse models (5, 6). Moreover, Urbanska et al. proposed that CFAP43 also plays a role in ciliary beating. Their study on *Tetrahymena thermophila* described a novel ciliary complex composed of two conserved WD-repeat proteins, Fap43 (the ortholog of human CFAP43) and Fap44, located near the two-headed inner dynein arm, IDA I1. Loss of these proteins altered ciliary waveform, beat stroke, and reduced swimming speed (9). Additionally, Rachev et al. demonstrated that Cfap43 modulates ciliary beating in mouse and Xenopus, and that loss of *Cfap43* leads to mucociliary clearance defects and hydrocephalus in addition to known male infertility (10).

While CFAP43 protein is implicated in both ciliary and flagellar structure and function, reported cases with *CFAP43* genetic variations have not typically exhibited clinical manifestations of ciliary dysfunction in the airways. In our study, both patients exhibited symptoms consistent with respiratory ciliary dysfunction, including chronic sinusitis and chronic wet cough since early infancy. Additionally, chest CT scan revealed bronchiectasis, providing further evidence of ciliary impairment. Notably, previous reports have documented a similar clinical presentation, wherein variations in genes initially associated with MMAF, such as *SPEF2* and *CFAP47*, were identified as the underlying cause for both ciliary and flagellar phenotypes (11, 12). These reports indicate that defects in some MMAF genes may cause a phenotypic spectrum ranging from MMAF without respiratory manifestations to infertile patients with PCD-like symptoms.

In conclusion, this study identified a novel variation in the *CFAP43* gene in two siblings presenting with MMAF and chronic respiratory symptoms. While our findings provide preliminary evidence suggesting a potential role of *CFAP43* mutations in both reproductive and respiratory phenotypes, further functional studies are needed to validate the direct effect of the identified variant on protein function and confirm its pathogenicity. Additionally, further research is warranted to identify other disease-causing genetic variations that may contribute to the MMAF phenotype with impaired motile cilia.

## Data Availability

De-identified summary data supporting the conclusions of this study are provided as Supplementary Table 2.

## Supplementary material

Supplementary Figure 1 Exome data analysis filtering approach. Illustration of the stepwise variant filtration approach used in the exome analysis of two affected siblings (Patient 1 and Patient 2). MAF, minor allele frequency; MS, missense; FS, frameshift; NFS, non-frameshift; SS, splice site; NS, nonsense.

Supplementary Table 1 Details of the final list of homozygous candidate variants shared by the two siblings after applying the whole exome sequencing filtering strategy. AA, amino acid; gnomAD, genome aggregation database; OMIM, online Mendelian inheritance in man; N/A, not available.

Supplementary Table 2

Comprehensive list of all variants identified in known MMAF and PCD genes in the two affected siblings, regardless of predicted pathogenicity.
