# Supplementary material for: Expanding the phenotypic spectrum associated with CFAP43 mutations: a case report of familial male infertility with respiratory manifestations
Source: Front Reprod Health. 2025 Nov 18;7:1609938. doi: 10.3389/frph.2025.1609938 (PMC12669162; doi:10.3389/frph.2025.1609938)
Supplement: Supplementary Table 1 — Details of the final list of homozygous candidate variants shared by the two siblings after applying the whole exome sequencing filtering strategy. AA, amino acid; gnomAD, genome aggregation database; OMIM, online Mendelian inheritance in man; N/A, not available. [file Presentation1.pptx]

## Slide 1
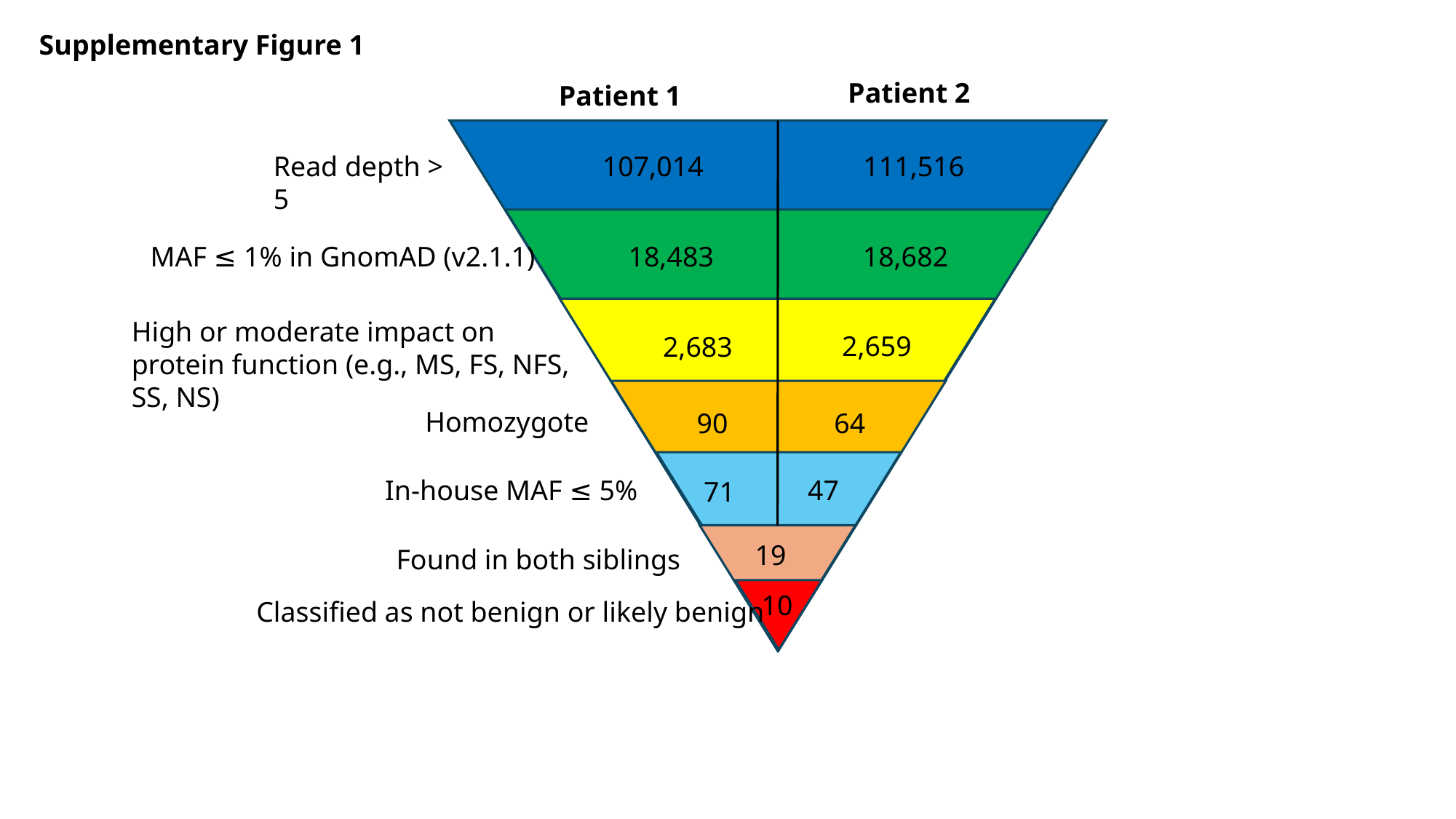

Supplementary Figure 1
Patient 2
Patient 1
Read depth > 5
107,014
111,516
MAF ≤ 1% in GnomAD (v2.1.1)
18,483
18,682
High or moderate impact on protein function (e.g., MS, FS, NFS, SS, NS)
2,659
2,683
Homozygote
90
64
In-house MAF ≤ 5%
47
71
19
0
Found in both siblings
10
Classified as not benign or likely benign

## Slide 2
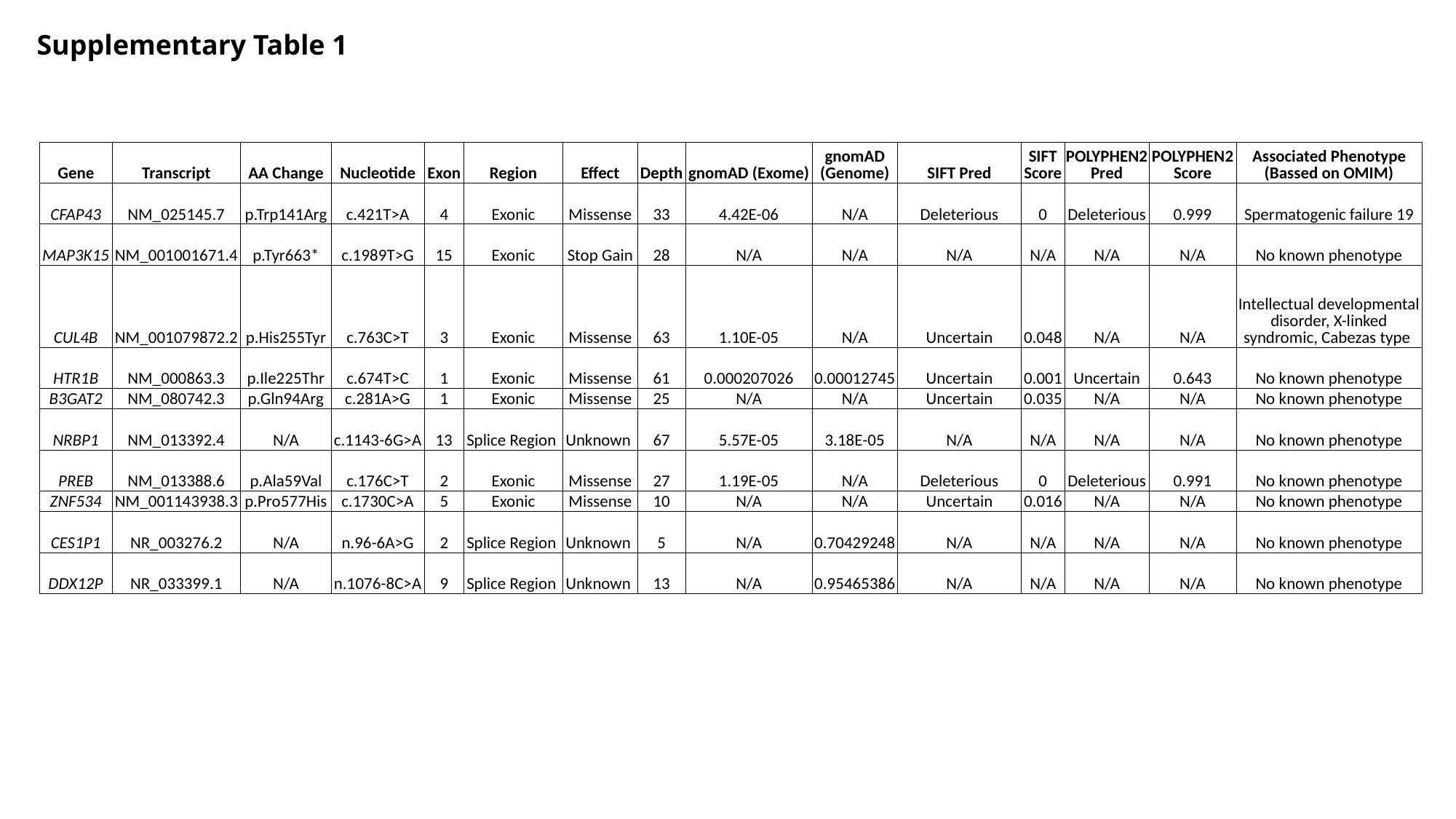

Supplementary Table 1
| Gene | Transcript | AA Change | Nucleotide | Exon | Region | Effect | Depth | gnomAD (Exome) | gnomAD (Genome) | SIFT Pred | SIFT Score | POLYPHEN2 Pred | POLYPHEN2 Score | Associated Phenotype (Bassed on OMIM) |
| --- | --- | --- | --- | --- | --- | --- | --- | --- | --- | --- | --- | --- | --- | --- |
| CFAP43 | NM\_025145.7 | p.Trp141Arg | c.421T>A | 4 | Exonic | Missense | 33 | 4.42E-06 | N/A | Deleterious | 0 | Deleterious | 0.999 | Spermatogenic failure 19 |
| MAP3K15 | NM\_001001671.4 | p.Tyr663\* | c.1989T>G | 15 | Exonic | Stop Gain | 28 | N/A | N/A | N/A | N/A | N/A | N/A | No known phenotype |
| CUL4B | NM\_001079872.2 | p.His255Tyr | c.763C>T | 3 | Exonic | Missense | 63 | 1.10E-05 | N/A | Uncertain | 0.048 | N/A | N/A | Intellectual developmental disorder, X-linked syndromic, Cabezas type |
| HTR1B | NM\_000863.3 | p.Ile225Thr | c.674T>C | 1 | Exonic | Missense | 61 | 0.000207026 | 0.00012745 | Uncertain | 0.001 | Uncertain | 0.643 | No known phenotype |
| B3GAT2 | NM\_080742.3 | p.Gln94Arg | c.281A>G | 1 | Exonic | Missense | 25 | N/A | N/A | Uncertain | 0.035 | N/A | N/A | No known phenotype |
| NRBP1 | NM\_013392.4 | N/A | c.1143-6G>A | 13 | Splice Region | Unknown | 67 | 5.57E-05 | 3.18E-05 | N/A | N/A | N/A | N/A | No known phenotype |
| PREB | NM\_013388.6 | p.Ala59Val | c.176C>T | 2 | Exonic | Missense | 27 | 1.19E-05 | N/A | Deleterious | 0 | Deleterious | 0.991 | No known phenotype |
| ZNF534 | NM\_001143938.3 | p.Pro577His | c.1730C>A | 5 | Exonic | Missense | 10 | N/A | N/A | Uncertain | 0.016 | N/A | N/A | No known phenotype |
| CES1P1 | NR\_003276.2 | N/A | n.96-6A>G | 2 | Splice Region | Unknown | 5 | N/A | 0.70429248 | N/A | N/A | N/A | N/A | No known phenotype |
| DDX12P | NR\_033399.1 | N/A | n.1076-8C>A | 9 | Splice Region | Unknown | 13 | N/A | 0.95465386 | N/A | N/A | N/A | N/A | No known phenotype |
